# Assessing the Diversity and Distribution of Apicomplexans in Host and Free-Living Environments Using High-Throughput Amplicon Data and a Phylogenetically Informed Reference Framework

**DOI:** 10.3389/fmicb.2019.02373

**Published:** 2019-10-23

**Authors:** Javier del Campo, Thierry J. Heger, Raquel Rodríguez-Martínez, Alexandra Z. Worden, Thomas A. Richards, Ramon Massana, Patrick J. Keeling

**Affiliations:** ^1^Department of Botany, University of British Columbia, Vancouver, BC, Canada; ^2^Department of Marine Biology and Ecology, Rosenstiel School of Marine and Atmospheric Science, University of Miami, Miami, FL, United States; ^3^Soil Science Group, CHANGINS, University of Applied Sciences and Arts Western Switzerland, Nyon, Switzerland; ^4^Department of Biosciences, Living Systems Institute, College of Life and Environmental Sciences, University of Exeter, Exeter, United Kingdom; ^5^GEOMAR – Helmholtz Centre for Ocean Research Kiel, Kiel, Germany; ^6^Department of Marine Biology and Oceanography, Institut de Ciències del Mar (CSIC), Barcelona, Spain

**Keywords:** apicomplexans, diversity, distribution, phylogeny, classification, metabarcoding, environmental sequencing, reference database

## Abstract

Apicomplexans are a group of microbial eukaryotes that contain some of the most well-studied parasites, including the causing agents of toxoplasmosis and malaria, and emergent diseases like cryptosporidiosis or babesiosis. Decades of research have illuminated the pathogenic mechanisms, molecular biology, and genomics of model apicomplexans, but we know little about their diversity and distribution in natural environments. In this study we analyze the distribution of apicomplexans across a range of both host-associated and free-living environments. Using publicly available small subunit (SSU) rRNA gene databases, high-throughput environmental sequencing (HTES) surveys, and our own generated HTES data, we developed an apicomplexan reference database, which includes the largest apicomplexan SSU rRNA tree available to date and encompasses comprehensive sampling of this group and their closest relatives. This tree allowed us to identify and correct incongruences in the molecular identification of apicomplexan sequences. Analyzing the diversity and distribution of apicomplexans in HTES studies with this curated reference database also showed a widespread, and quantitatively important, presence of apicomplexans across a variety of free-living environments. These data allow us to describe a remarkable molecular diversity of this group compared with our current knowledge, especially when compared with that identified from described apicomplexan species. This is most striking in marine environments, where potentially the most diverse apicomplexans apparently exist, but have not yet been formally recognized. The new database will be useful for microbial ecology and epidemiological studies, and provide valuable reference for medical and veterinary diagnosis especially in cases of emerging, zoonotic, and cryptic infections.

## Introduction

Protistan parasites account for >10% of the World Organization for Animal Health’s list of notifiable diseases of terrestrial and aquatic animals (Stentiford et al., 2014) and 25% of the major groups of pathogens that cause animal and plant species extinction and extirpation (Fisher et al., 2012). From an ecological perspective, they play key roles in the regulation and structure of natural communities in different environments (Preston et al., 2016). Recent environmental microbial surveys also show that putatively parasitic protists are abundant in soils, freshwater, and marine systems (de Vargas et al., 2015; Geisen et al., 2015; Simon et al., 2015; Mahé et al., 2017).

Among these parasites, Apicomplexa stands out. Apicomplexans are parasites that infect a wide variety of animals and are morphologically distinguished by the presence of an apical complex, a suite of structures used to invade host cells (Votýpka et al., 2016). Most apicomplexans also possess a relict plastid, the apicoplast (McFadden et al., 1996), which is non-photosynthetic but essential for parasite survival (McFadden et al., 2017). Well-known human parasites include *Toxoplasma* (Tenter et al., 2000), *Cryptosporidium* (Checkley et al., 2015), and the malaria agent *Plasmodium* (Keeling and Rayner, 2015). Other apicomplexans are poorly studied, even though they are diverse and widespread in the environment and are hypothesized to play key roles in ecosystem function (de Vargas et al., 2015; Mahé et al., 2017). Of these, the gregarines are the largest but mostly understudied group of putative invertebrate parasites (Leander, 2008), also associated with juvenile frogs (Chambouvet et al., 2016). While the medical importance of apicomplexans is well-known, recent high-throughput environmental sequencing surveys (HTESs) have shown a high diversity and abundance of apicomplexans in marine and terrestrial environments, suggesting our understanding of the impact of parasitic eukaryotes is probably underestimated. In the Tara Oceans global marine survey, apicomplexans represent the third most represented group of amplicons from parasites (following the Marine Alveolate groups I and II) (de Vargas et al., 2015). In soils, apicomplexans are also highly represented in amplicon data, representing >50% of operational taxonomic units (OTUs) in tropical forest soils (Mahé et al., 2017).

Understanding what this diversity and distribution means requires a more detailed dissection of which apicomplexans appear in which environments. This is currently not possible because we lack a robust phylogenetic framework (e.g., a reference tree) upon which to base such inferences. Moreover, it has recently been shown that the apicomplexans are the sister group to another odd collection of microbial predators (colpodellids) and putatively symbiotic algae (chromerids), collectively known as chrompodellids or “Apicomplexan-related lineages” (ARLs) (Leander et al., 2003; Moore et al., 2008; Oborník et al., 2012; Woo et al., 2015). These lineages have aided in understanding of how apicomplexans evolved to become parasites and the ecological conditions that might have led to this transition.

Most HTES studies use the small subunit (SSU) of the ribosomal RNA (18S rRNA for eukaryotes and 16S rRNA for prokaryotes) to identify/barcode the members of the targeted community. There are two major reference databases available for the 18S rRNA (Quast et al., 2012; Guillou et al., 2013). Both draw most of their taxonomic information directly from GenBank (Balvočiūtë and Huson, 2017), which contains a significant number of misidentified sequences, likely a historical and systematic problem derived from incomplete databases available when sequences are deposited (del Campo et al., 2018). In one database, PR2, a significant number of groups have been curated by experts, but the phylogenetic framework for apicomplexans is under-developed, and in other databases, e.g., SILVA, there is minimal evaluation of the data as the data processing is automated (Cavalier-Smith, 2014; Janouškovec et al., 2015). The propagation of misidentified sequences often affects subsequent identifications, and ecological inferences more broadly, but in the case of pathogenic species (including apicomplexans) has also led to misdiagnosis of infections in humans (Yuan et al., 2012).

Therefore, while we know from environmental survey data that apicomplexans and their relatives are widespread and diverse, no further interpretation is possible without a phylogenetically informed understanding of their sequence diversity. With that in mind, here we describe a comprehensive phylogenetic framework of all the apicomplexan and ARL sequence diversity, including those not identified, initially misidentified from all current isolates and environmental studies. We manually annotated the taxonomic information for each sequence using phylogenetic trees and curated associated information (such as the isolation source, origin, etc.) from the literature. As a product of this process, we propose several changes to apicomplexan classification, correcting the affiliation of several organisms, and describing a dozen new groups. Using this reference framework as a tool, we examined the distribution of apicomplexans and ARLs in environmental data (both publicly available data and new data sets described here), covering environments from soils to the ocean, from sediments to the water column, at a level of taxonomic detail previously unachieved. Apicomplexans are shown to be present in all the environments examined, and more diverse than previously reported. This diversity was suspected but could not be detected using current databases and because of limited sampling. Overall, these data represent a major tool for understanding the diversity and distribution of what are perhaps one of the most globally abundant animal parasites and highlight potential roles they might play in trophic networks in soils and marine systems.

These data also contribute to an overall understanding of the parasitic nature of the group. Most available literature on apicomplexans define them as parasites. That has been shown for members of the better-known groups like coccicians or hematozoans, but has not formally been tested for most apicomplexans, especially the widespread and diverse gregarines. Most gregarines are nevertheless described as being parasitic because they were isolated from animal hosts, but in most of the cases Koch’s postulates have never been proven (Fredricks and Relman, 1996). As large surveys based on molecular data become increasingly dominant in our view of microbial distribution, this problem becomes increasingly important because, for example, with HTES data we have fewer direct observations of the context of a host–microbe interaction. The level of apicomplexan diversity we document is incompatible with formal proofs of Koch’s postulates, further elevating the importance of clearly and accurately documenting patterns and correlations that will help to interpret the enormous amount of data generated from genomics and environmental genomics.

## Results

### A Reference Tree for Apicomplexans

**FIGURE 1:**
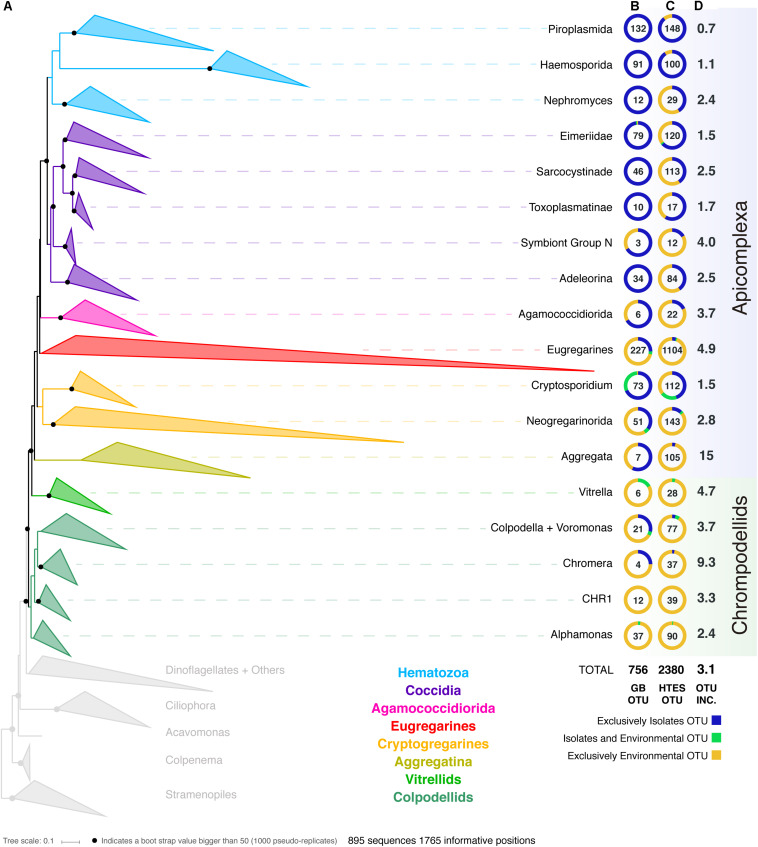
**(A)** Apicomplexan reference tree inferred from a maximum-likelihood (RAxML) phylogenetic tree constructed (best tree of 1000) using 18S rRNA sequences. Black dots represent bootstrap values >50% (1000 bootstrap replicates). The tree has been collapsed in the main apicomplexan groups based on our taxonomic annotation (Supplementary Table S1). The tree showing all the OTUs (97% similarity clustering) is available as Supplementary Information S1. The first two columns next to the Apicomplexan groups’ names inform about the origin (colored circle) and the number of OTUs (number in the middle of the circle) retrieved from **(B)** GenBank and from **(C)** high-throughput environmental sequencing (HTES) studies. The HTES OTUs have been added to the reference tree using the Evolutionary Placement Algorithm in RAxML and the correspondent tree is available as Supplementary Information S2. The last column indicates the fold increase in numbers of OTUs after adding the HTES OTUs to the reference tree. In the figure “Isolates” stands for sequences retrieved from cultured or isolated organisms while “Environmental” stands for OTUs retrieved from culture and isolation independent molecular surveys. CHR1 corresponds to a group of Colpodellids constituted of exclusively environmental sequences.

In order to improve our understanding of the molecular diversity of the apicomplexans, we constructed a 18S rRNA tree including all apicomplexan sequences retrieved from GenBank based on similarity and no shorter than 500 bp, resulting in a dataset which consists of 8,392 sequences representing a total of 756 distinct OTUs at 97% identity (Figure 1). From this curated phylogeny, we identified 12 novel environmental clades above the genus level within the apicomplexans, and 4 within the chrompodellids (Supplementary Information S1 and Supplementary Table S1). These clades consisted entirely of environmental sequences and included no known identified species. Additionally, the relationships identified between some well-studied groups also required alterations to the current taxonomic nomenclature. This was particularly clear for the genera *Babesia* and *Theileria*, where we identified five groups that had been identified as either *Babesia* or *Theileria*, but were not phylogenetically classifiable as being related to the type species. Both genera have minor SSU rRNA variants, but these tend to cluster with the major SSU rRNA variant from the same genus (Bhoora et al., 2009). There is no indication our results result from undetected paralogs, and these OTUs should therefore be re-examined as they most likely represent new genera (Supplementary Table S1). Similar situations were found for deep-branching eimeriids and, less surprisingly, for gregarines, a group already known to be highly diverse and undersampled. It is worth mentioning that the archigregarines, one of the previously established order of gregarines based on morphology (Leander, 2008), could not be recovered monophyletically in our studies and their members are included in the eugregarines.

We then added all reads corresponding to apicomplexans and related lineages to this curated tree, incorporating >3.5 million sequences from HTES studies derived from diverse environments. We recovered a total of 2,380 apicomplexan-related OTUs at 97% similarity (increasing the number of apicomplexan and ARL OTUs by a factor of 3). The environmental sequences were not evenly distributed in the apicomplexan tree, but novel environmental diversity did appear in clades dominated by both clinical isolates and also groups that have been previously reported only in environmental surveys. In all cases, except the piroplasmids, the addition of HTES sequence data dramatically increased the proportion of OTUs retrieved from the environment compared with those from clinical isolates going from 270 to 1799 (Figure 1). Overall, 30 new apicomplexan groups could be identified using HTES data, most of them within coccidians (Supplementary Table S2).

Along with the sequence data, we also curated all available metadata; either using information provided with the sequence accession, or by cross-referencing sequences with information in the publications describing the sequences. For clinical isolates this consisted of information such as host species or tissue type, while for environmental sequences it included location and the nature of the environment. Most of the publicly available sequences had associated metadata, and at the broadest level these corresponded to our expectations: most hematozoans and coccidians derived from isolates, while the rest of the apicomplexan groups were better represented in environmental surveys, particularly the eugregarines. In the case of the chrompodellids, most of the OTUs were also retrieved exclusively from environmental surveys, since there are relatively few cultures of these organisms.

### A Reference Database for Apicomplexans and ARLs

We used the sequences from our comprehensive apicomplexan SSU tree to build a reference database that can be used to assign HTES reads an identity by sequence similarity and also to infer certain information regarding their environmental and host distribution. As for the tree the reference database consisted only of sequences longer than 500 bp (consistent with the previous analysis of 8,392 sequences). For each sequence in the database we provided an accession number and we manually assigned a phylogenetically informed taxonomic string based on the tree that consists of six ranks below Eukarya and started with Alveolata. Right after the taxonomic string we included a column that corresponded to the name of the sequence. The name is either derived from a proper species name or a strain, but alternatively could also be a clone name in the case of sequences generated from environmental clone libraries. While the taxonomic string was assigned to each accession number based on the reference tree generated, the sequence names remained untouched for the purposes of identification within GenBank and the literature. Right after the taxonomic information we added the environmental metadata fields, starting with the origin of the sequence, if it came from an environmental clone library or from an isolate. After that, two columns with environmental information, named Environment 1 and Environment 2, with 1 being the most inclusive and 2 more detailed. More than 90% of the sequences had this field filled with 166 unique values for the less inclusive one. The next column was the geo-localization field, which was available for close to 90% of the sequences and populated by 715 unique values. The very last field was the host taxonomy string field. The string consisted of five taxonomic ranks, Phylum, Class, Order, Family, and Species. Sixty-four percent of the sequences contained information regarding the host taxonomy, using >700 unique terms at the species level. The metadata information was initially automatically retrieved from GenBank and after that double-checked with the literature that was also used to fill the significant gaps of the GenBank available metadata.

**FIGURE 2:**
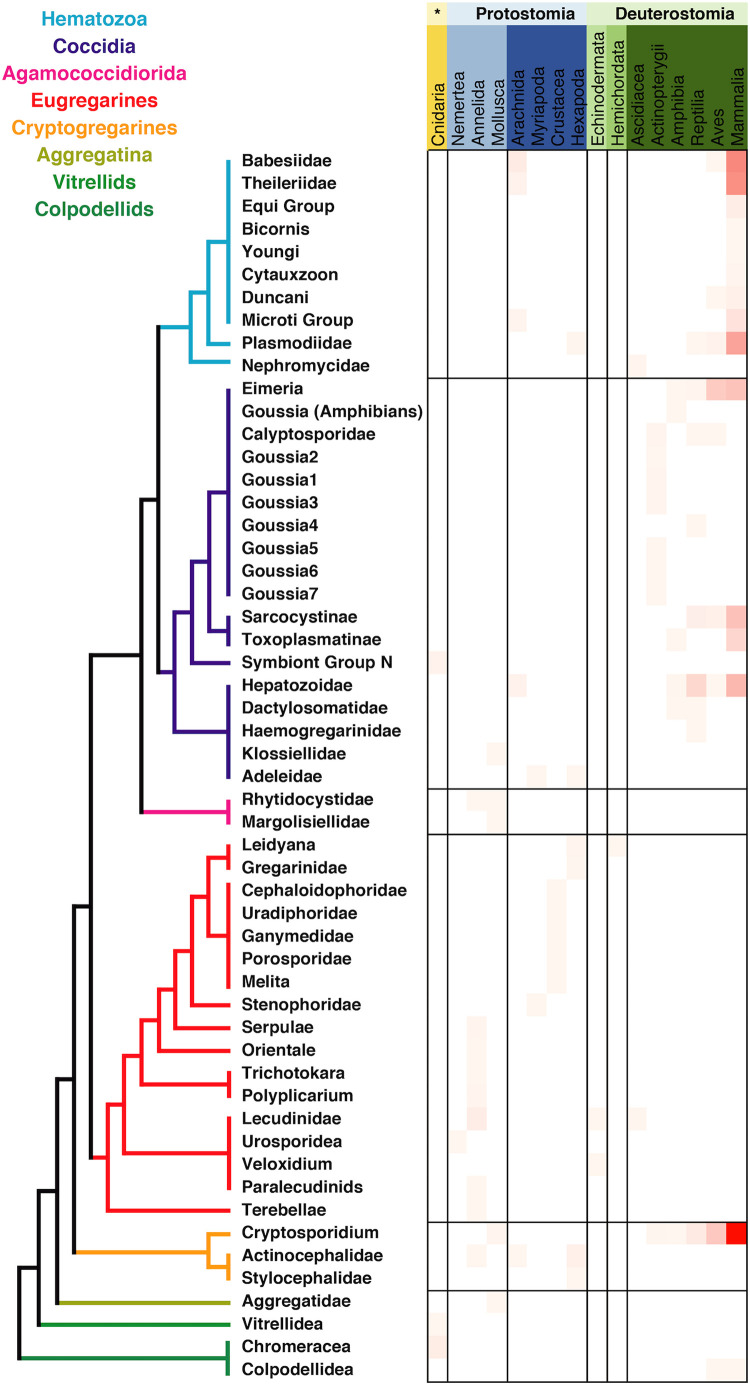
Apicomplexan host distribution heatmap based on the information associated to sequences available in GenBank. Detailed information on the taxonomic annotation of the apicomplexan sequences used in this figure is available in Supplementary Table S1.

The metadata associated with the sequences retrieved from isolates was in most of the cases congruent with the information regarding tissue localization and host distribution of the correspondent organisms. When looking at the host distribution of the SSU rRNA sequences retrieved from GenBank (Figure 2) we observed that in terms of distribution certain groups like the Agamoccidioria, the gregarines, and Aggregata showed up mostly within the invertebrates, whereas coccidians, hematozoa, and cryptosporidium were obtained mostly from mammals. Considering the messiness of this kind of data in GenBank, it was a positive sign that at least for Apicomplexans this information could be reused and represented a good starting point to build a reliable database, especially if the information was double-checked and the gaps were filled using literature searches.

### Environmental Distribution

**FIGURE 3:**
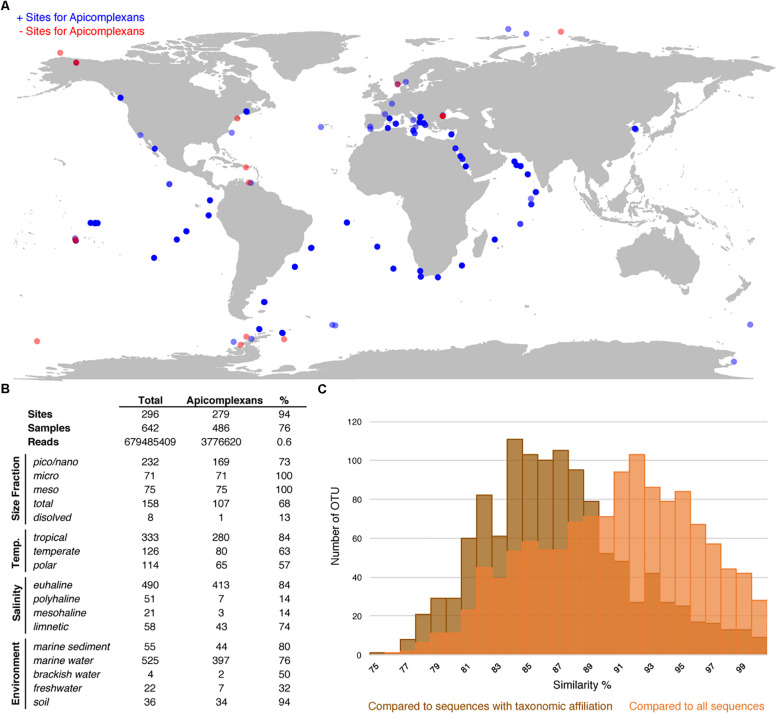
**(A)** World map showing all the HTES analyzed sites, in blue the sites where apicomplexan reads have been reported in red those in which no apicomplexan reads have been found. **(B)** Distribution (presence) of apicomplexans in the analyzed HTES datasets as a whole and clustered by different features. Size fraction (pico/nano: 0.8–20 μm, meso: 20–180 μm, micro: 180–2000 μm, total: no size fractioning, dissolved: dissolved DNA), temperature (tropical: >18°C, temperate: 10–18° C, polar: <10°C), salinity (euhaline: 30–40 PSU, polyhaline: 18–30 PSU, mesohaline: 5–18 PSU, limnetic: <0.5 PSU). **(C)** Blast similarity against GenBank of the HTES apicomplexan reads. In brown the results when comparing against sequences annotated with taxonomic affiliation and in orange the results when comparing against all the apicomplexan sequences including those environmental sequences that do not correspond to any identified apicomplexan species.

To evaluate the utility of the reference tree and database, and, at the same time study the environmental distribution of apicomplexans in existing environmental datasets, we analyzed 642 environmental surveys from 296 locations across different environments, including soil, freshwater, and marine habitats (Figures 3A,B). We retrieved apicomplexans from 76% of the samples and 94% of the environment types, and found apicomplexans represented 0.6% of the amplicons as a whole. Overall, apicomplexans and their related lineages appeared to have a higher relative abundance in tropical than polar waters, and in marine and soil amplicon data as opposed to freshwater environments (Figure 3B). Comparing the apicomplexan sequences in HTES data with all available sequences in GenBank showed that the sequence similarity between retrieved reads and all other available sequences peaks around 93%, but when the comparison was made only against described species the peak drops to 84–85% (Figure 3C). This suggests that the vast majority of environmental sequences from apicomplexans come from yet-to-be identified species.

**FIGURE 4:**
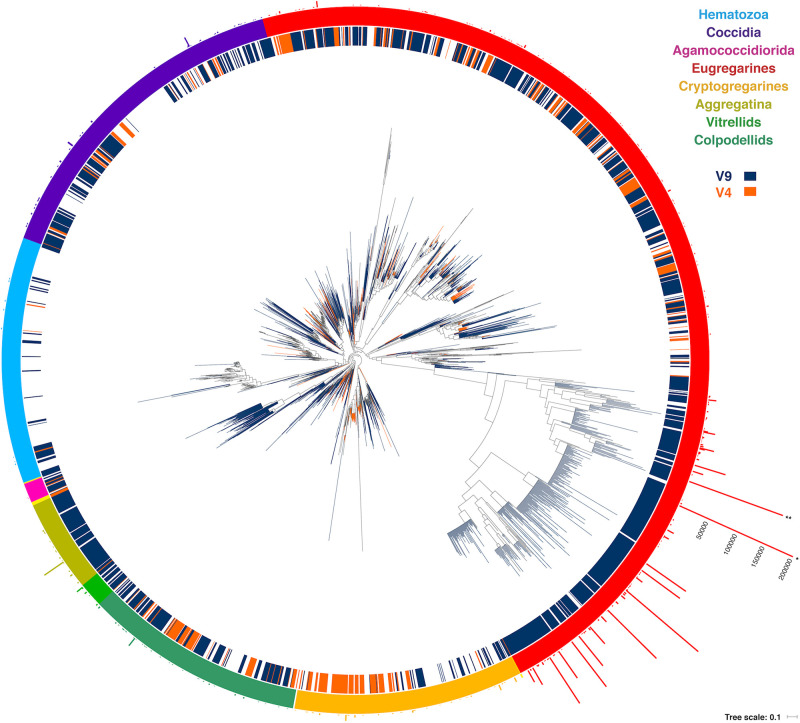
Round tree showing the placement of the short reads using the Evolutionary Placement Algorithm in RAxML (EPA-RAxML) on the reference tree. The colors of the leaves and the inner crown surrounding the tree indicate the 18S rRNA region of the corresponding OTUs (V4 or V9). The outer crown indicates the number of reads per OTU.

One might expect environmental samples to yield mostly sequences associated with free-living clades, such as predatory chrompodellids, but we found the opposite. Most of the reads identified as apicomplexans from environmental surveys fell within the eugregarines, which is perhaps not surprising since they are known to be diverse and are less sampled than other apicomplexan groups. The gregarine life cycle also involves releasing large numbers of resistant, infective cells into the environment, rather than direct, host-to-host infection, which might also be expected to lead to high relative abundance representation in environmental data. Eugregarine amplicon abundance was closely followed by that of the comparatively well-studied coccidians, where a substantial environmental diversity of adeleorinids, sarcocystids, and basal *Goussia*-like eimerids, and agreggadids was identified (Figure 4). We also retrieved sequences corresponding to cryptogregarines and more surprisingly, hematozoans. Sequences belonging to the chromopodellids were also identified, including both members of clades containing free-living genera like *Alphamonas*, *Voromonas*, or *Colpodella*, as well as genera thought to be symbionts such as *Chromera* and *Vitrella*. There were no significant differences between the distribution of 18S rRNA V4 and V9 reads across the tree that cannot be explained by the source of the sequences.

**FIGURE 5:**
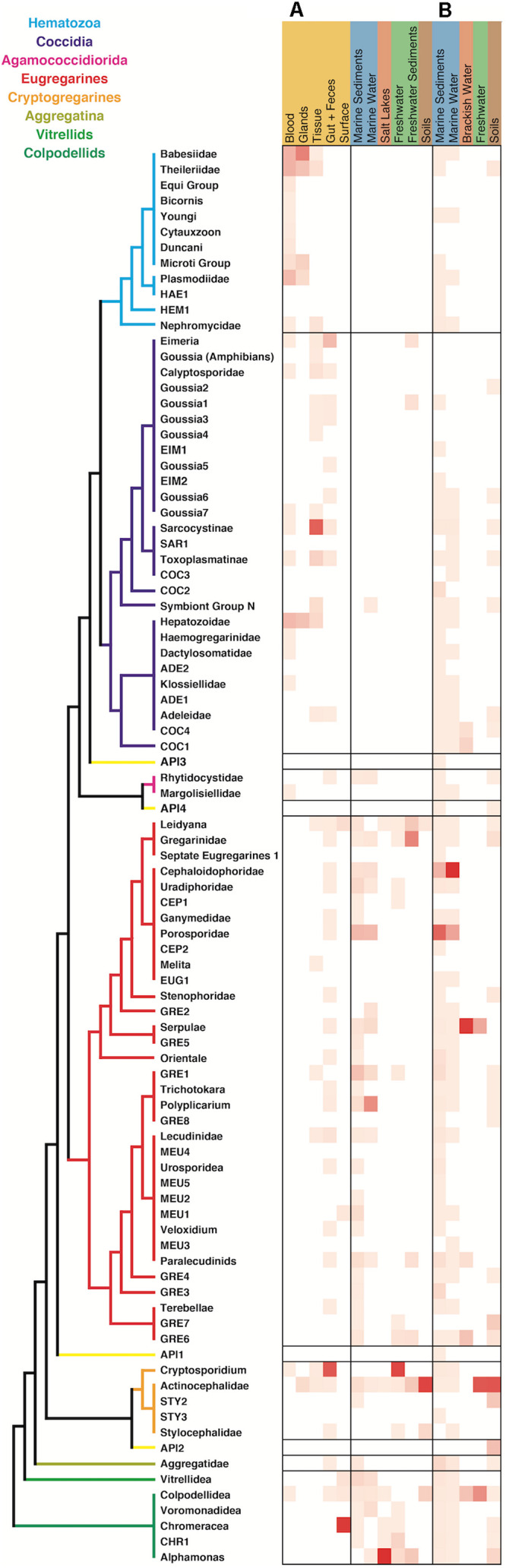
Apicomplexan environment distribution heatmap based on the information associated to the sequences retrieved from **(A)** GenBank and **(B)** relative amplicon abundances in HTES studies. The novel lineages obtained only by HTES studies are inserted on the tree shown in Figure 2, respecting the original topology. Detailed information on the taxonomic annotation of the apicomplexan sequences used in this figure is available in Supplementary Table S1.

When comparing the environmental distribution using both Sanger and HTES sequences (Figure 5), we observed that hematozoans and coccidians were commonly retrieved from clinical isolates but rarely observed in Sanger environmental clone libraries. However, they did appear in marine, freshwaters, and soils HTES datasets. We retrieved OTUs from well-known groups and also from 11 novel clusters that could not be assigned to any of the described hematozoans or coccidians. In the case of the eugregarines and cryptogregarines, the increase in diversity from HTES data was extreme: members of both groups have been retrieved from isolates in the past, but because nearly all inhabit invertebrate cells relatively few have been previously characterized. The addition of HTES data confirmed the abundant representation of eugregarines and cryptogregarines in the environment and showed that they had the highest relative abundances among amplicons along with being the most widespread and diverse.

In marine systems the Cephaloidophoridea and Porosporidae eugregarines had the highest relative abundances among apicomplexans, while in freshwaters and soils the Actinocephalidea cryptogregarines had the highest relative abundances. In the marine environment, the most highly represented groups in open ocean were the Cepholoidophoridae and Porosporidae gregarines, while in coastal environments the Lecudinidae, GRE1, and Chromareraceae dominated the apicomplexan-derived amplicons (Supplementary Figure S1). Cepholoidophoridae and Porosporidae were the most highly represented in epipelagic and benthic environments, while Dactylosomatidae, Klossiellidae, Adeleidae, and Gregarinidae were the most common in mesopelagic and bathy/abyssopelagic environments. Most of the apicomplexan groups were reported from oxic environments, but a few were also identified in anoxic ones, where Dactylosomatidae, Klossiellidae, and Gregarinidae showed the highest amplicon abundances.

## Discussion

### A Framework for Biomedical and Ecological Studies

The widespread use of HTES data to infer characteristics about the distribution and ecology of microbial life relies entirely on the quality of the reference database to translate the catalog of sequences recovered from an environment into accurate taxonomic identifications of the organism from which they are derived. Despite their importance, available databases have not been phylogenetically curated for most protist lineages. A common practice is instead to export annotations from GenBank, where many sequences are mis-annotated or not annotated at all (del Campo et al., 2018). As a result, GenBank is the *de facto* reference database for users who may be unaware of how to access specific references databases.

In the case of the apicomplexans, the problems with the current state of our reference data are clear from comparisons of our manually curated reference database and tree with existing GenBank annotation. There are extensive mistakes in some human and animal parasites, like *Theileria* and *Babesia* (Schnittger et al., 2012) at the genus level (Supplementary Table S1), or in *Cryptosporidium* and eimeriids at the species level (Supplementary Table S1). Mis-annotation is even more common in the eugregarines and the cryptogregarines at various taxonomic levels, and mistaken affiliations are relatively common across the entire tree of apicomplexans (Supplementary Table S1). Putting this in a biomedical or veterinarian context, an incorrect annotation of a genetic barcode in a reference database can lead to misdiagnosis and potentially ineffective treatments. For ecological studies the situation is much worse, because the environmental sequences in GenBank are usually not annotated taxonomically at all, so even a perfect sequence match leads to no information about identity. To give an example of how this leads to problems, if one looks at the protists reported in the Tara Oceans survey of marine microbes, they retrieve from their data a widespread presence of the malaria parasite in marine samples (de Vargas et al., 2015). Specifically, the Tara Oceans analysis recovered 1,123 OTUs representing 293,824 reads out of 6 million apicomplexan reads that were widespread across the photic zone, and these were assigned as *Plasmodium falciparum*. Analyzing the same data using our phylogenetically curated reference framework, we have retrieved only two OTUs representing a total of nine Tara Oceans reads that are phylogenetically affiliated with *Plasmodium*: this is three orders of magnitude fewer *Plasmodium* OTUs and five orders of magnitude fewer reads (Supplementary Table S2). The large number of “*Plasmodium*” reads that were mis-identified using existing reference databases are in fact mostly Cephaloidophoridea, gregarine parasites of crustaceans (see below): the ecological implications of this misidentification need no elaboration.

### Diversity and Distribution of Clinically Important Apicomplexans

The publicly available information in GenBank is an obvious reflection of the biases in apicomplexan research foci, documenting mostly human and cow parasites. Thus, GenBank cannot offer any aid to our understanding of lineage distributions (Figure 2 and Supplementary Table S1). The biological diversity of apicomplexans as inferred from HTES data is dominated by gregarines, particularly the eugregarines, of which nearly all known species inhabit invertebrates. The biases in GenBank are even more prominent when we examine the similarity between reference sequences and HTES sequences, which on average share only 85% similarity to identified species. This value is extremely low, and indicates that not only has the majority of apicomplexan diversity not been characterized at the molecular level, but that we lack even representatives of many major lineages for comparison. A large-scale screen of inverebrate-associated apicomplexans would greatly help to improve our knowledge about the diversity, the ecology, and the evolution of the apicomplexans, because we have barely begun to scratch the surface of this part of the apicomplexan tree.

Within the cultured apicomplexans, there is an obvious bias in favor of clinically important species as well. Among these clinical isolates, blood borne pathogens from mammals (and particularly from bovids and humans) are dominant, and there are also significant numbers of *Cryptosporidium* isolates from guts and feces: all reflecting the interest of the medical and veterinary communities. Interestingly, most of these well-known pathogens (with the exception of *Cryptosporidium*) are not retrieved from Sanger clone library environmental sequence surveys, but they do appear in HTES data, especially from soils and marine samples. Nevertheless, the phylogenies of well-known pathogens are significantly improved by analyzing the data as a whole, including all the other apicomplexans and both sequences from isolates and from environmental surveys. As mentioned above, *Babesia* and *Theileria* are paraphyletic, suggesting the need for a taxonomic revision of these groups. Similarly, *Cryptosporidium* isolates are taxonomically problematic at the species level, and at some level so is *Toxoplasma*.

The presence of sequences from important animal- and human-parasites in the free-living environment can also help us to identify unsuspected reservoirs, or even novel parasite diversity. Biomedical research tends to focus on strains already important to human disease, not unreasonably, but it would also be useful to examine HTES datasets more carefully to identify the natural distribution of apicomplexan lineages of clinical and veterinary interest outside these hosts, since these might reveal potentially undescribed sources of parasite transmission and propagation.

### The Environmental Distribution of the Apicomplexans and Related Lineages

Apicomplexans appear in all environments examined, as has already been suggested by previous analyses (de Vargas et al., 2015; Mahé et al., 2017). There appears to be a bias toward marine and soil samples, but this may be due to a bias in the number of samples coming from these environments as brackish or freshwater environments are relatively under-studied (Figure 3), and they are not rare in samples that do exists from such environments. Their broad distribution and high abundance both suggest apicomplexans play a significant role in food webs and the population structure of their animal hosts.

When looking more closely at the distribution by group, the relative amplicons abundances of eugregarines suggest that they are the most abundant apicomplexans, that they are the most diverse (at least for the variable regions of the 18S rRNA gene that have been studied), and also have the widest spatial distributions (Figures 4, 5). The neogregarines are particularly well-represented among apicomplexan amplicons in soils, but not as diverse as eugregarines. Other groups that stand out are the adeleorinids, the eimeriids, and the sarcocystids, which are mostly present in marine samples. Adeleorinids includes some described genera that are extremely highly represented, like *Klossia*, which infects mollusks (Barta et al., 2012), or fish parasites in the Dactylosomatidae (Barta, 1991), which are the most prevalent apicomplexan amplicons in the deep ocean and also anoxic environments (Supplementary Figure S1). In the case of the eimeriids, most of the sequences fall at the base of the group in a paraphyletic assemblage including the fish-parasite *Goussia* (Jirků et al., 2002). Fish parasites have also been described among sarcocystids and other coccidians (Davies and Ball, 1993; Davies, 1995), but little is known about their biology or diversity, and virtually nothing from a molecular perspective. Most of the coccidian sequences come from the sediment, and based on their known host distribution it seems plausible that this signal corresponds to the spores of fish parasites (López-García et al., 2003).

In other cases, observed distribution patterns are more surprising, in particular, the apicomplexan known as “symbiont N or corallicolids” (Kwong et al., 2019) and the apicomplexan-related genera *Vitrella* and *Chromera*. All these groups have been described as coral symbionts (Janouškovec et al., 2013) or associated to coral reefs (Mathur et al., 2018) and, although we do not have samples from any coral environment in our dataset, sequences from all three were retrieved in our analyses. Finding such sequences from sediments may simply raise the possibility that these organisms associate with other anthozoans and not just corals, but we also identified them in water column samples. This may be due to capturing infective stages between hosts, may suggest that these organisms are not necessarily symbionts, or that closely related sisters are free-living. Most strangely, however, reads associated with all three groups of coral symbionts were also retrieved from soils. This is much harder to rationalize with their being coral symbionts and indicates that some of the biological diversity of these groups has not yet been explored.

A similar situation is surprisingly seen in the hematozoa. Hematozoans are best known for infecting terrestrial animals, but 2,483 hematozoan amplicons (Supplementary Table S2) were retrieved from marine samples, predominantly sediments (Figure 5). Half of these belong to the ascidian symbiont, *Nephromyces*, which is not unexpected since this is a marine host, but most other reads were closely related to well-known blood borne parasites best known from non-marine hosts, like *Babesia* or *Plasmodium*. Neither Haemosporidia nor Piroplasmida are not known to infect fish, and this together with the relatively low representativeness of these reads suggests alternative origins such as marine birds, where these parasites have been previously reported (Quillfeldt et al., 2010).

### Gregarines as the Most Abundant Apicomplexans and Their Putative Roles in the Environment

Eugregarines and neograrines are the most highly represented apicomplexans in amplicon data from marine and soil samples, respectively. This is not surprising because they are largely invertebrate-associated, and invertebrates represent the majority of animals, both taxonomically and numerically (Wilson, 1987). These two groups of apicomplexans are poorly studied, but it seems likely that some of the more common but unidentified gregarines are associated with zooplanktonic and meiofaunal animals, which play crucial roles in the food webs (and so too, by extension, would their associated microeukaryotes). The role of gregarines as pathogens or parasites is still debated and there is every reason to expect some variation in their relationship to their hosts. Gregarines are known to cause disease in shrimps (Jones et al., 1994) and in insects they are involved in the decrease of their host fitness by tissue damage, reducing their body size, fecundity, and longevity (Sulaiman, 1992; Valigurová and Koudela, 2005; Valigurová, 2012). In most species, however, their pathogenicity has not been examined specifically.

The most abundant apicomplexan group in our dataset is the Cephaloidophoridea, which also represent one of the most abundant OTUs in Tara Oceans (where, as noted above, they are mis-identified as *Plasmodium*) (de Vargas et al., 2015). Cephaloidophoridea gregarines infect crustaceans from both Malacostraca and Maxillopoda (Jones et al., 1994; Rueckert et al., 2011; Figure 2), and are known to infect copepods (Théodoridès, 1989). Considering the size fractions in our marine water samples (from 0.8 to 2000 μm), and the reported abundance of Cephaloidophoridea in previous publications, it seems likely that they infect marine zooplankton in large numbers. Another group of eugregarines, the Porosporidae, also infect crustaceans, including copepods (Rueckert et al., 2011). This group of eugregarines are phylogenetically related to the Cephaloidophoridea and were also commonly retrieved from both the water column and sediments. Both these groups may therefore play a major role in the marine food webs by regulating copepods populations, which are themselves the most prominent members of the zooplankton and a key link between phytoplankton and fish larvae (Humes, 1994). Examining the co-occurrence of these gregarines with members the zooplankton could allow this hypothesis to be tested, while screening copepod individuals in the wild would be needed to conclusively confirm whether they are infected with these common but unidentified Cephaloidophoridea and Porosporidae, and whether the apicomplexans cause disease or death of the hosts.

In soils, the neogregarines are the most abundantly represented group in apicomplexan amplicon data, with the Actinocephalidae standing out. Related organisms are commonly retrieved from bees (Plischuk et al., 2011), fleas (Alarcón et al., 2011), and earthworms (Field and Michiels, 2005). Apart from the difficulty of defining what is and what is not a pathogen, there are also infections that we do not know which disease they cause, if any. In the case of earthworms, *Monocystis* is an extremely common apicomplexan, with 100% infection rates in certain communities (Field and Michiels, 2005). Earthworms play a significant role in the soil food web, where they are responsible of organic-matter breakdown, nutrient enrichment, particles relocation, and the dispersal of microorganisms, altogether shaping the soil structure and physic-chemical properties. In the case of *Monocystis*, there is no clear evidence it has a significant ecological impact, but it has been shown to be mildly deleterious to host fitness (Field and Michiels, 2005), so could only play a subtler role in host population structure, which in turn could be significant. Apicomplexans more broadly have been retrieved in high densities in tropical soils, with eugregarines and neogregarines dominating (Mahé et al., 2017). As with the ocean, some of these gregarines must be relevant to regulating host population diversity and activities that affect soil structure and composition. Such conclusions await concrete identification of apicomplexan–host associations as well as determining whether infection leads to disease or death, and the database has provided the first step of identifying which parasites to focus on.

Apart from a putative role as host-regulators, the wide distribution and high representativity of apicomplexans suggests they may also represent an alternative heterotrophic pathway for transferring carbon within the trophic web. Symbionts’ consumption rates are high in the environment, and this consumption is frequent, non-accidental, and influences food web properties (Johnson et al., 2010). On the other hand, when parasites infect their hosts they have access to more organic matter than free-living heterotrophic species that depend on prey encounters (Worden et al., 2015). Overall, apicomplexans, and eugregarines and neogregarines in particular, might make a significant impact on food web dynamics and the carbon cycle in marine and soil systems simply through their heterotrophic activities, and not just on how they change host population numbers and structure.

## Conclusion

Gregarines (eugregarines and neogregarines) were identified as the most abundantly represented and widespread apicomplexans in our analyses. Considering that previous studies have shown that the apicomplexans are also well-represented at the amplicon level compared with the rest of the micro-eukaryotes, gregarines as putative invertebrate parasites have the potential to play an important role regulating the meiofaunal and zooplanktonic communities in soil and marine systems, directly impacting the carbon cycle. It will be important to determine exactly the functional role that gregarines play in these environments, and its impact, by examining patterns of host–parasite population change together with more direct observations of their interactions. Only then can apicomplexans be realistically integrated into models for marine and soil trophic networks.

High-throughput environmental sequencing metabarcoding has been an extremely useful tool for microbial ecology, and recently eDNA metabarcoding is becoming standard for rapid screening of organismal diversity in conservation (Bohmann et al., 2014) and environmental monitoring (Pawlowski et al., 2016), providing a useful tool for diagnosis. However, it is crucial to have reliable reference databases to accurately identify the sequences generated through HTES. Our study provides the necessary tools to study the diversity and ecological distribution of the apicomplexans and establishes the basis to use the 18S rRNA gene as a reliable biomarker to detect apicomplexans in host associated and free-living environments, and by extension its use in epidemiology or diagnosis. Having this reference framework, consisting on a tree and a reference database, is the only way to interpret such data in a useful and comparable way.

We have shown the suitability of the aforementioned approach and tools by analyzing a large quantity of HTES data from public sources and generated *de novo*. Using the described framework, we have shown that apicomplexans are diverse and widespread based on their amplicon distributions in the environment. We cannot directly infer their organismal abundance using our dataset, but based on previous publications (de Vargas et al., 2015; Mahé et al., 2017) they are one of the most highly represented parasites in terms of 18S rRNA amplicon relative abundances, particularly in soils and marine systems. The novel diversity revealed here includes unrecognized parasites of humans and a range of ecologically and commercially important animals, in addition to several potential emergent pathogens. From a veterinary and medical perspective, it would be interesting to use eDNA techniques in the future to explore the prevalence of apicomplexans in the environment and target potential sources of infection.

## Materials and Methods

### Sample Processing and Sequence Generation

Soil samples were obtained during summer from Calvert and Hecate islands on the central coast of British Columbia, Canada. A total of 36 samples were stored in coolers containing ice packs and afterward frozen at −80°C within 6 h. DNA extraction of the samples was performed with the FastDNA SPIN Kit for soil (MP Biomedicals, Solon, OH, United States). Protist communities were investigated using high-throughput Illumina sequencing on the hypervariable V4 region of the 18S rRNA gene using using the Phusion^®^ High-Fidelity DNA Polymerase (Thermo Fisher, MA, United States) and the general eukaryotic primer pair TAReuk454FWD1 and TAReukREV3 (Stoeck et al., 2010). Paired-end sequencing of the library was performed with the Illumina MiSeq platform using the MiSeq Reagent v3 chemistry (Illumina, San Diego, CA, United States). The library was 300 bp paired-end sequenced at the Genotyping Core Facility of the University California Los Angeles (Los Angeles, CA, United States). Further details on sampling and sequence generation can be found in Heger et al. (2018). Amplicon data are available on NCBI Sequence Read Archive (SRA) under project number: PRJNA396681 and have been also used for a previous publication from our group (Heger et al., 2018).

Marine sediment samples (Supplementary Table S3) were taken on-board of the MBARI research vessel *Western Flyer* in the North Pacific Ocean (Monterey Canyon) and preserved with RNA Lifeguard (Qiagen, CA, United States). From each sampling core ∼12 g of sediment was transferred into a 50 mL falcon tube, using sterilized spatula in laminar flow hood. Samples were placed immediately in a −80°C freezer on board. RNA extraction was performed using the Qiagen PowerSoil RNA isolation kit (Qiagen, CA, United States), using the DNase treatment described in the protocol. RNA quality and quantity for samples was checked using a 2100 Bioanalyzer (Agilent Technologies). Each RNA sample was then reverse transcribed into cDNA using SuperScript III reverse transcriptase (Invitrogen, CA, United States) with random hexamers. Respective negative controls were done during the process. Protist communities were investigated using high-throughput Illumina sequencing on the hypervariable V9 region of the 18S rRNA gene using the Phusion^®^ High-Fidelity DNA Polymerase (Thermo Fisher, MA, United States) and the general eukaryotic primer pair 1380F and 1510R (Amaral-Zettler et al., 2009). Paired-end sequencing of the library was performed with the Illumina HiSeq 2000 platform (Illumina, San Diego, CA, United States) with NEXTflex DNA sequencing kits and an identifying NEXTflex DNA barcode with 8-base indices (Bioo Scientific, TX, United States). The library was 150 bp paired-end sequenced at the Exeter Sequencing Service (University of Exeter, United Kingdom). Amplicon data are available on NCBI SRA under project number: PRJNA521526.

### Reference Phylogenetic Tree and Database

All GenBank SSU rDNA sequences identified as Apicomplexans or Chrompodellids were retrieved using the corresponding taxid (5794/877183, 177937, and 333132). Sequences shorter than 500 bp were excluded. The remaining sequences were clustered at 97% identity using USEARCH v7.0.1090 (Edgar, 2010). In order to build the tree, 22 other alveolates and 18 sequences were used as outgroups. All sequences were aligned and trimmed using MAFFT 7 with default settings (Katoh and Standley, 2013) and trimAl (Capella-Gutiérrez et al., 2009), respectively. A maximum-likelihood phylogenetic tree was constructed with RAxML 8.1.3 (Stamatakis, 2014) using the rapid hill climbing algorithm and GTRCAT evolutionary model. Whether sequences belong to the Apicomplexans and the Chrompodellids was determined based on the tree topology and literature. Verified sequences were then used to iteratively retrieve more sequences from GenBank using blastn (Camacho et al., 2009) against nt as previously described (del Campo and Massana, 2011; del Campo and Ruiz-Trillo, 2013) in order to enrich the tree with environmental sequences or sequences with a wrong taxid that were not recovered in the first place. Putative chimeric sequences were manually examined, and the final retrieved dataset was clustered as well at 97% in order to build a tree. The final phylogenetic tree was built using RAxML with the settings mentioned above. Statistical support for the consensus tree was calculated using non-parametric bootstrapping with 1,000 replicates.

In order to construct a reference database sequences from isolates were initially annotated based on previously published works. We adopted the established taxonomy as our default classification method when possible (Cavalier-Smith, 2014; Adl et al., 2019). For groups containing isolates with no formal taxonomic affiliation assignable based on the tree an informal name for the group has been provided based on the genus, species name of associated metadata. In the case of groups containing only environmental representatives a group named using three letters and a number has been provided. Metadata for the sequences in our dataset was downloaded from GenBank using custom scripts. For sequences still missing environmental data, their information was then collected manually from the literature.

### Analysis of HTES Sequences

Sequences annotated as Alveolates were retrieved from three publicly available 18S rRNA datasets, VAMPS, BioMarKs, and Tara Oceans (Huse et al., 2014; de Vargas et al., 2015; Massana et al., 2015) and two additional datasets generated by us. Overall the analyzed data cover a wide range of environments from soils and freshwater to the sunlit ocean and the deep-sea sediments (up to 3000 m). The analyzed dataset contains both V4 and V9 region reads and several size fractions. The fasta file containing all reads was sorted by length using USEARCH and clustered into OTUs with 97% similarity using QIIME with default setting (UCLUST). OTUs were then aligned with the reference alignment using PyNAST (Caporaso et al., 2010a) embedded in QIIME (Caporaso et al., 2010b) (align_seqs.py). The reference alignment was the same alignment that was used to generate the reference phylogenetic tree. OTUs that the PyNAST algorithm failed to align were discarded. The PyNAST alignment output was merged with the reference alignment and filtered for gap positions using QIIME (filter_alignment.py) with gap filtering threshold set to 0.99 and entropy threshold set to 0.0001. Identification of Apicomplexans and Chrompodellids reads used a maximum-likelihood phylogenetic approach by mapping the OTUs onto our reference tree using the Evolutionary Placement Algorithm (EPA) of RAxML (Berger and Stamatakis, 2011). OTUs that were not placed within the Apicomplexans and Chrompodellids were removed. Trees using the remaining sequences were built consecutively until no more reads were placed outside our two groups of interest. OTUs and their clustered sequences were then annotated according to their placement. For novel groups containing only short reads we adopted the same annotation as for the environmental exclusive groups retrieved from GenBank. OTUs that were not placed with any previously defined groups were assigned a new name as outlined above. The annotated OTU table and corresponding sample metadata (Supplementary Information S3,S4) were processed for community analysis using QIIME.

## Data Availability Statement

The datasets generated for this study can be found in NCBI Sequence Read Archive, PRJNA521526 and PRJNA396681. The reference database generated in this study and provided in Supplementary Table S1 has also been deposited in PR2 (Guillou et al., 2013) and it is available at https://github.com/pr2database/pr2database. PR2 will also host future updates of this reference database.

## Author Contributions

JC and PK designed the study. JC, TH, RR-M, AW, TR, RM, and PK obtained the samples. JC performed the analyses and wrote the manuscript, with input from all authors. PK supervised the work.

## Conflict of Interest

The authors declare that the research was conducted in the absence of any commercial or financial relationships that could be construed as a potential conflict of interest.

## Supplementary Material

FIGURE S1 Apicomplexan environment distribution heatmap based on the information associated to the sequences retrieved from HTES studies for different environmental features: redox state, open ocean vs. coastal, depth, size fraction, and temperature.

TABLE S1 Apicomplexan reference database built from 18S rRNA sequences bigger than 500 bp retrieved from GenBank. The database contains the following columns: AN, sequence accession number; Columns 0–6, taxonomic string; Name, name given to the sequence in GenBank; Origin, isolate or environmental sequence as defined in Figure 1; Environment 1, environmental feature; Environment 2, environmental feature; Location, geographical feature; Host Phylum–Class–Order–Family–Species, host taxonomic information when available; Sequence, corresponding 18S sequence; Size, sequence base pairs size.

TABLE S2 Taxonomic summary of the HTES reads at the genus level.

TABLE S3 Depth and coordinates for the Monterey Bay deep-sea sediment samples.

INFORMATION S1 Apicomplexan 18S rRNA (sequence length > 500 bp) reference tree inferred from a maximum-likelihood (RAxML) phylogenetic tree (best tree of 1000 and 1000 bootstrap replicates).

INFORMATION S2 Apicomplexan 18S rRNA EPA-RAxML tree using the HTES reads as query and the Apicomplexan 18S rRNA reference as backbone.

INFORMATION S3 Apicomplexans HTES reads OTU table.

INFORMATION S4 Apicomplexans HTES reads mapping file.
