# Corrigendum: Assessing the Diversity and Distribution of Apicomplexans in Host and Free-Living Environments Using High-Throughput Amplicon Data and a Phylogenetically Informed Reference Framework

**DOI:** 10.3389/fmicb.2020.576322

**Published:** 2020-10-08

**Authors:** Javier del Campo, Thierry J. Heger, Raquel Rodríguez-Martínez, Alexandra Z. Worden, Thomas A. Richards, Ramon Massana, Patrick J. Keeling

**Affiliations:** ^1^Department of Botany, University of British Columbia, Vancouver, BC, Canada; ^2^Department of Marine Biology and Ecology, Rosenstiel School of Marine and Atmospheric Science, University of Miami, Miami, FL, United States; ^3^Soil Science Group, CHANGINS, University of Applied Sciences and Arts Western Switzerland, Nyon, Switzerland; ^4^Department of Biosciences, Living Systems Institute, College of Life and Environmental Sciences, University of Exeter, Exeter, United Kingdom; ^5^GEOMAR – Helmholtz Centre for Ocean Research Kiel, Kiel, Germany; ^6^Department of Marine Biology and Oceanography, Institut de Ciències del Mar (CSIC), Barcelona, Spain

**Keywords:** apicomplexans, diversity, distribution, phylogeny, classification, metabarcoding, environmental sequencing, reference database

In the original article, the reference for Kotabová et al., 2012 was incorrectly written as Kotabová, E., Vancová, M., Lukeš, J., Oborník, M., Modri, D., Lukeš, M., et al. (2012). Morphology, ultrastructure and life cycle of *Vitrella brassicaformis* n. sp., n. gen., a novel chromerid from the great barrier reef. *Protist* 163, 306–323. doi: 10.1016/j.protis.2011.09.001. It should be Oborník, M., Modrý, D., Lukeš, M., Cernotíková-Stríbrná, E., Cihlár, J., Tesarová, M., et al. (2012). Morphology, ultrastructure and life cycle of *Vitrella brassicaformis* n. sp., n. gen., a novel chromerid from the great barrier reef. *Protist* 163, 306–323. doi: 10.1016/j.protis.2011.09.001 Furthermore, the reference should be cited as Oborník et al. 2012.

In the original article Moore, R. B., Oborník, M., Janouškovec, J., Chrudimský, T., Vancová, M., Green, D. H., et al. (2008). A photosynthetic alveolate closely related to apicomplexan parasites. *Nature* 451, 959–963. doi: 10.1038/nature06635 was not cited in the article. The citation has now been inserted in INTRODUCTION, Paragraph 3 and should read:

Understanding what this diversity and distribution means requires a more detailed dissection of which apicomplexans appear in which environments. This is currently not possible because we lack a robust phylogenetic framework (e.g., a reference tree) upon which to base such inferences. Moreover, it has5 recently been shown that the apicomplexans are the sister group to another odd collection of microbial predators (colpodellids) and putatively symbiotic algae (chromerids), collectively known as chrompodellids or “Apicomplexan-related lineages” (ARLs) (Leander et al., 2003; Moore et al., 2008; Oborník et al., 2012; Woo et al., 2015). These lineages have aided in understanding of how apicomplexans evolved to become parasites and the ecological conditions that might have led to this transition.

The authors apologize for these errors and state that this does not change the scientific conclusions of the article in any way. The original article has been updated.
