# Supplementary material for: Assessing the Diversity and Distribution of Apicomplexans in Host and Free-Living Environments Using High-Throughput Amplicon Data and a Phylogenetically Informed Reference Framework
Source: Front Microbiol. 2019 Oct 23;10:2373. doi: 10.3389/fmicb.2019.02373 (PMC6819320; doi:10.3389/fmicb.2019.02373)
Supplement: FIGURE S1 — Apicomplexan environment distribution heatmap based on the information associated to the sequences retrieved from HTES studies for different environmental features: redox state, open ocean vs. coastal, depth, size fraction, and temperature. [file Data_Sheet_1.PDF]

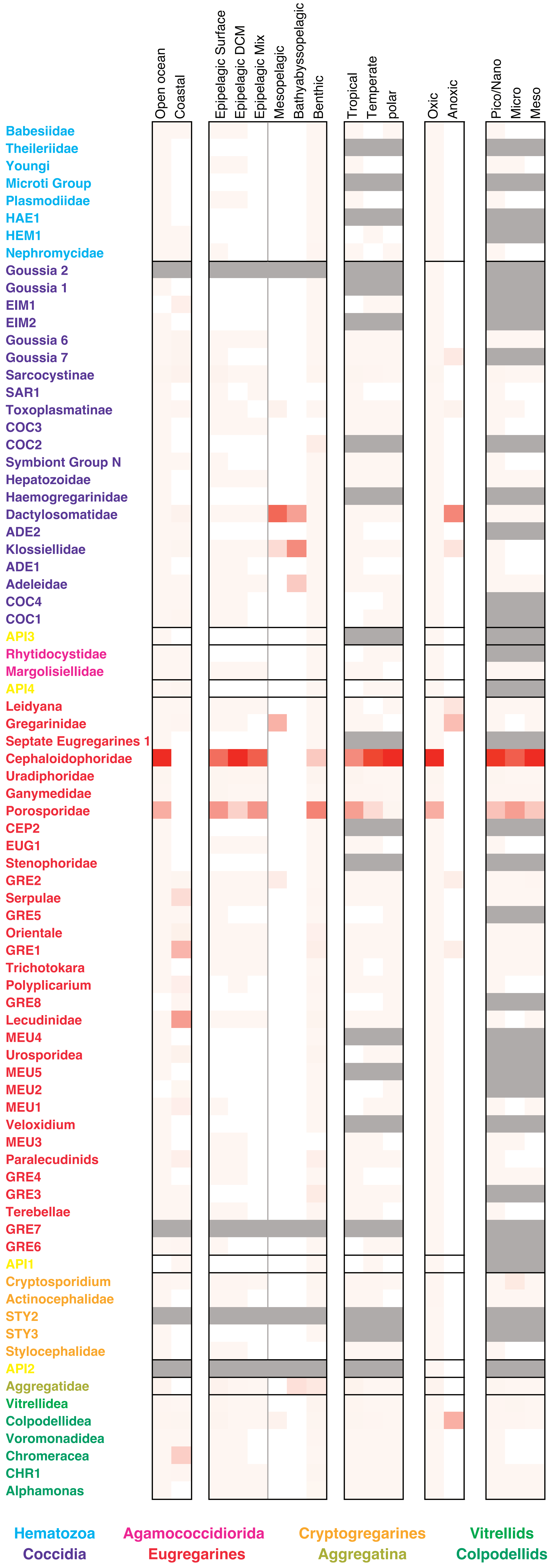

Open ocean
Coastal
Epipelagic Surface
Epipelagic DCM
Epipelagic Mix
Mesopelagic
Bathyabyssopelagic
Benthic
Tropical
Temperate
polar
Oxic
Anoxic
Pico/Nano
Micro
Meso
Babesiidae
Theileriidae
Youngi
Microti Group
Plasmodiidae
HAE1
HEM1
Nephromycidae
Goussia 2
Goussia 1
EIM1
EIM2
Goussia 6
Goussia 7
Sarcocystinae
SAR1
Toxoplasmatinae
COC3
COC2
Symbiont Group N
Hepatozoidae
Haemogregarinidae
Dactylosomatidae
ADE2
Klossiellidae
ADE1
Adeleidae
COC4
COC1
API3
Rhytidocystidae
Margolisiellidae
API4
Leidyana
Gregarinidae
Septate Eugregarines 1
Cephaloidophoridae
Uradiphoridae
Ganymedidae
Porosporidae
CEP2
EUG1
Stenophoridae
GRE2
Serpulae
GRE5
Orientale
GRE1
Trichotokara
Polyplicarium
GRE8
Lecudinidae
MEU4
Urosporidea
MEU5
MEU2
MEU1
Veloxidium
MEU3
Paralecudinids
GRE4
GRE3
Terebellae
GRE7
GRE6
API1
Cryptosporidium
Actinocephalidae
STY2
STY3
Stylocephalidae
API2
Aggregatidae
Vitrellidea
Colpodellidea
Voromonadidea
Chromeracea
CHR1
Alphamonas
Hematozoa
Coccidia
Agamococcidiorida
Eugregarines
Cryptogregarines
Aggregatina
Vitrellids
Colpodellids
